# The evolution of flexibility and function in the Fc domains of IgM, IgY, and IgE

**DOI:** 10.3389/fimmu.2024.1389494

**Published:** 2024-10-09

**Authors:** Rosaleen A. Calvert, Rosemary A. Nyamboya, Andrew J. Beavil, Brian J. Sutton

**Affiliations:** Randall Centre for Cell and Molecular Biophysics, Faculty of Life Sciences and Medicine, King’s College London, London, United Kingdom

**Keywords:** IgM, IgY, platypus IgE, human IgE, Fc region, flexibility, evolution, receptor

## Abstract

**Introduction:**

Antibody Fc regions harbour the binding sites for receptors that mediate effector functions following antigen engagement by the Fab regions. An extended “hinge” region in IgG allows flexibility between Fab and Fc, but in both the most primitive antibody, IgM, and in the evolutionarily more recent IgE, the hinge is replaced by an additional domain pair in the homodimeric six-domain Fc region. This permits additional flexibility *within* the Fc region, which has been exploited by nature to modulate antibody effector functions. Thus, in pentameric or hexameric IgM, the Fc regions appear to adopt a planar conformation in solution until antigen binding causes a conformational change and exposes the complement binding sites. In contrast, IgE-Fc principally adopts an acutely bent conformation in solution, but the binding of different receptors is controlled by the degree of bending, and there is allosteric communication between receptor binding sites.

**Methods:**

We sought to trace the evolution of Fc conformational diversity from IgM to IgE *via* the intermediate avian IgY by studying the solution conformations of their Fc regions by small-angle X-ray scattering. We compared four extant proteins: human IgM-Fc homodimer, chicken IgY-Fc, platypus IgE-Fc, and human IgE-Fc. These are examples of proteins that first appeared in the jawed fish [425 million years ago (mya)], tetrapod (310 mya), monotreme (166 mya), and hominid (2.5 mya) clades, respectively.

**Results and discussion:**

We analysed the scattering curves in terms of contributions from a pool of variously bent models chosen by a non-negative linear least-squares algorithm and found that the four proteins form a series in which the proportion of acutely bent material increases: IgM-Fc < IgY-Fc < plIgE-Fc < huIgE-Fc. This follows their order of appearance in evolution. For the huIgM-Fc homodimer, although none are acutely bent, and a significant fraction of the protein is sufficiently bent to expose the C1q-binding site, it predominantly adopts a fully extended conformation. In contrast, huIgE-Fc is found principally to be acutely bent, as expected from earlier studies. IgY-Fc, in this first structural analysis of the complete Fc region, exhibits an ensemble of conformations from acutely bent to fully extended, reflecting IgY’s position as an evolutionary intermediate between IgM and IgE.

## Introduction

**Figure 1:**
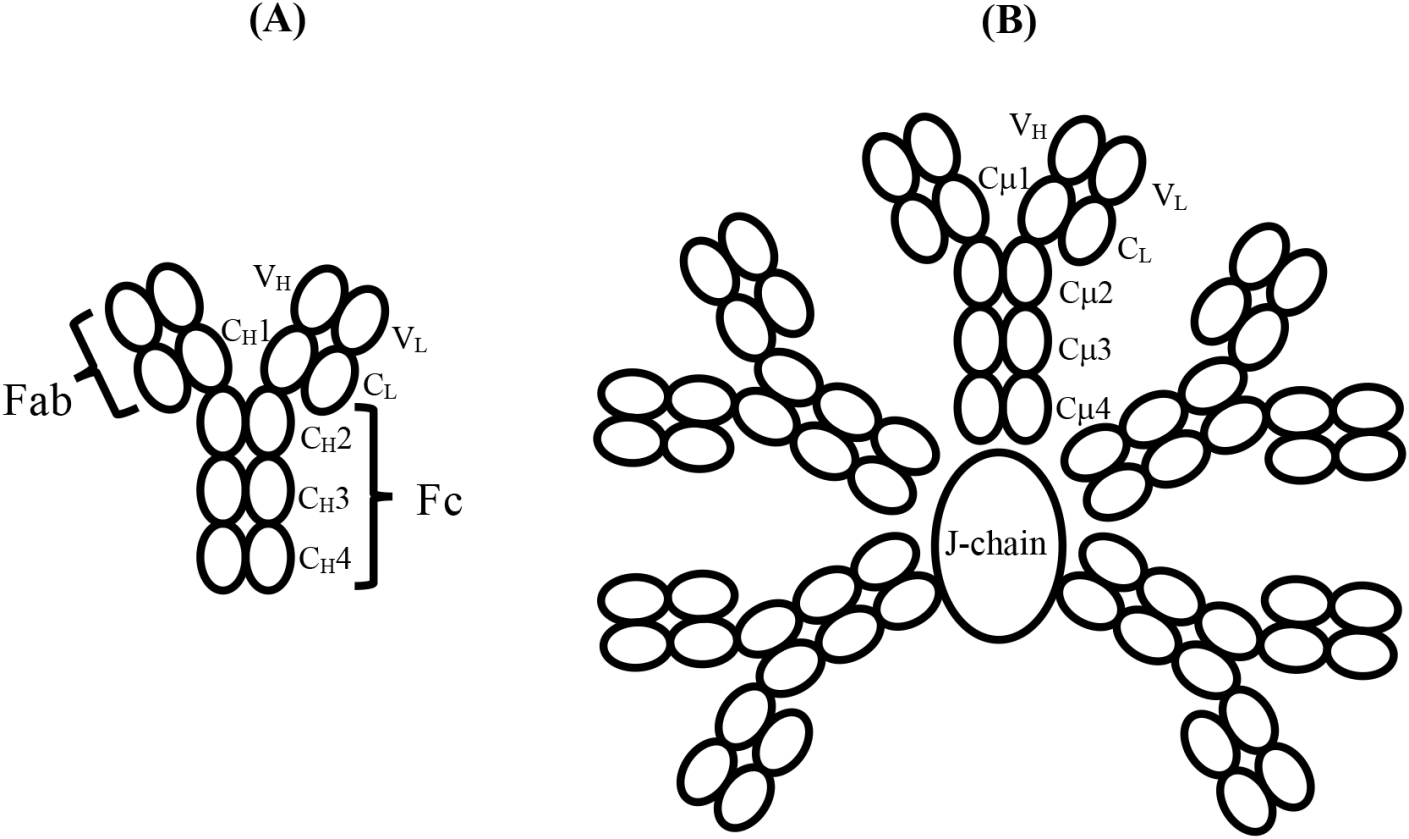
**(A)** Schematic structure and domain nomenclature for the heavy and light chains of an antibody such as IgY or IgE, which consists of six domains in the Fc region. The antigen-binding Fab region is also shown. **(B)** Schematic of the domain structure and nomenclature of pentameric IgM with the J-chain, in the absence of which, a hexamer of the H_2_L_2_ subunits forms. The IgM-Fc region of a single subunit, such as that indicated by the domain nomenclature, consisting of six domains, enables direct comparison with IgY-Fc and IgE-Fc. The complement binding sites in IgM lie in the Cμ3 domains close to the connection with the Cμ2 domains; they are not indicated here but are illustrated schematically in **Figure 2**. Disulphide bridges within and between subunits, and the tail-piece extensions of the H chains that engage with the J-chain, are not shown.

Throughout the evolution of antibodies from the emergence of IgM in jawed vertebrates 425 million years ago (mya) to the most recent antibody studied here, hominid IgE, which is just 2.5 mya, the homodimeric four-chain structure consisting of two identical heavy (H)-chains and two identical light (L)-chains has been preserved (**Figure 1A**). In this conventionally drawn “Y-shaped” structure, two Fab arms interact with antigen, and the Fc region mediates effector functions such as complement activation and binding to cell surface receptors. Although human IgM consists of either five or, less commonly, six of these H_2_L_2_ units covalently linked in a (pseudo) hexagonal array (**Figure 1B**), almost all other classes of antibodies function as a single homodimer. The Fc regions of IgM and IgE both consist of six H-chain domains, (C_H_2-C_H_3-C_H_4)_2_, as does the evolutionary precursor to mammalian IgE (and IgG), namely avian IgY (1–3), but in other classes of antibodies, such as IgG, IgA, and IgD, an extended “hinge” region replaces the C_H_2 domain; this permits greater flexibility between the Fab arms and the Fc region and is important for antigen binding. However, conformational flexibility *within* the Fc regions of IgM and IgE is known to be critical for expressing their effector functions, but in different ways for these two isotypes, and structures ranging from fully extended to acutely bent have been observed, as described below.

**Figure 2:**
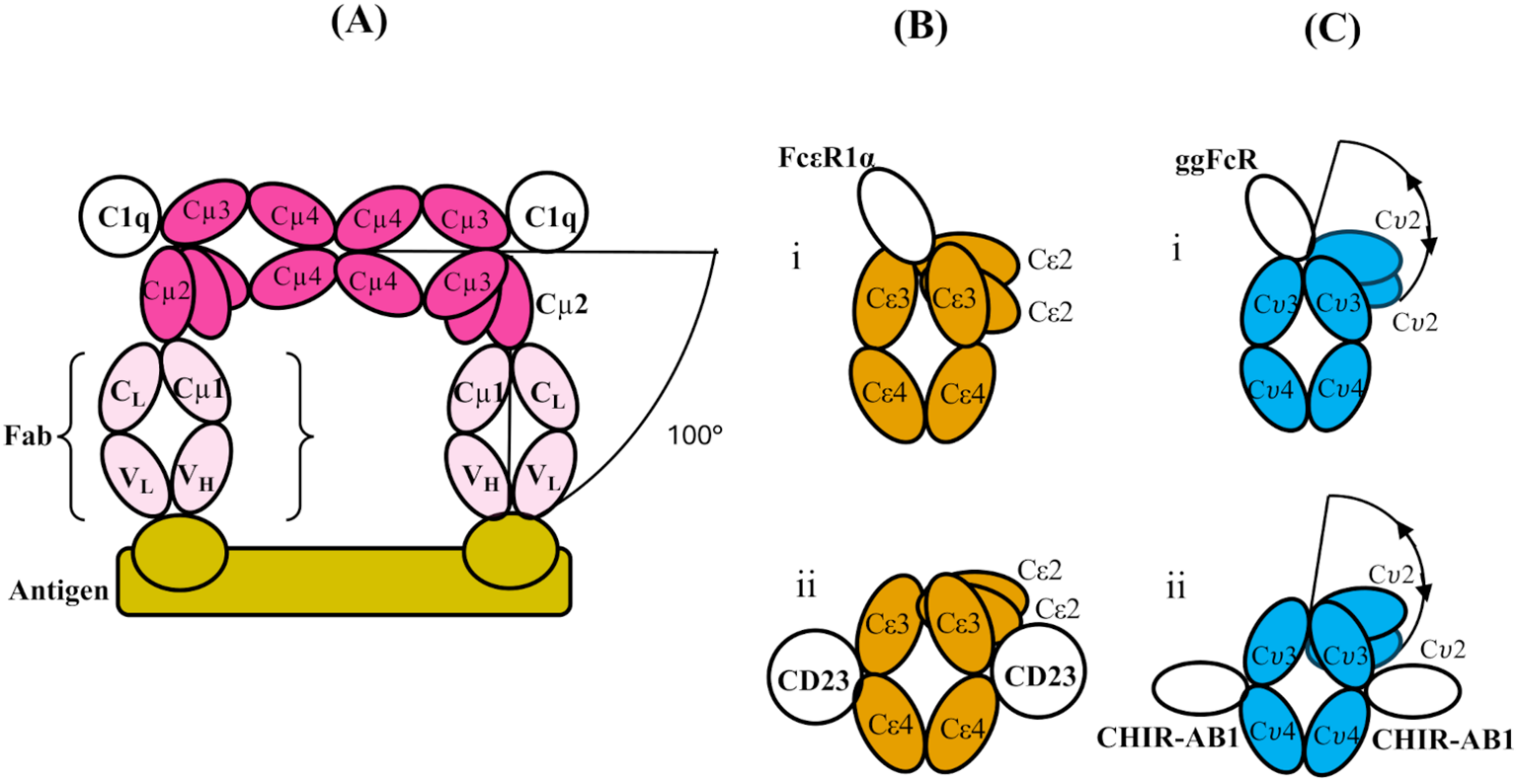
**(A)** Schematic structure of a part of two subunits of IgM (which themselves are part of a pentamer), bent between their Cμ2 and Cμ3 domains to allow two Fab regions to engage with a multi-valent antigen (bamboo colour). The figure is adapted from cryo-electron tomography structures presented in ref. (7). Only one Fab from each IgM subunit is shown (pale pink; the other is omitted for clarity), and the Fc regions (pink) of the two subunits are shown in contact through their Cμ4 domains (as shown schematically for the pentamer in **Figure 1B**). The bending of the Fc regions at the Cμ3-Cμ2 interface exposes binding sites for C1q head domains in the Cμ3 domains as indicated, and the overall bend of the Fab arms relative to the plane of the Fc_5_ region is 100°C. **(B)** Schematic illustrations of the domains of IgE-Fc when bound to (i) FcεRIα and (ii) CD23 (PDB 2Y7Q and PDB 4EZM, respectively). In both, there is an acute bend between the Cε2 and Cε3 domains, which is slightly more pronounced in the former and accompanied by an “opening” of the Cε3 domains. **(C)** Schematic illustration of the domain structure of IgY-Fc with the receptors i) ggFcR and ii) CHIR-AB1 bound. Note that the disposition of the Cυ2 domains is unknown in free or receptor-bound IgY-Fc, and the locations of the receptor sites are only implied by homology and mutagenesis evidence (refer to the text). The extent of any flexibility (indicated by the double-headed arrows) of the Cυ2 domains relative to the rest of the Fc is also unknown, prior to the present study.

Human IgM, free in serum in the absence of antigen, was shown in early studies to adopt a planar structure, with the Cμ2, Cμ3, and Cμ4 domains in an extended conformation (4, 5) (as shown schematically in **Figure 1B**). Both as a pentamer and (even more so) as a hexamer, IgM is a potent activator of complement through the classical pathway, but only in the presence of antigen. Electron micrographs of IgM in the presence of excess antigen show dislocation of the Fab arms from the central Fc disc to form “table” or “staple”-like structures, and these complexes activate complement *via* the classical pathway by binding of protein C1q (4), the first sub-component of the complement cascade. Feinstein suggested that the C1q-binding sites were obscured in the extended structure and only became accessible in the dislocated bent conformation (4), a proposition supported by later small-angle X-ray scattering (SAXS) analysis and molecular modelling (5). Whether the bend in the IgM-Fc was at the Cμ1-Cμ2 or Cμ2-Cμ3 junction was unclear, but the SAXS analysis suggested the latter (5). Two recent cryo-EM studies of serum IgM (in which the Cμ2 domains are visible), both alone (6) and in the presence of antigen and complement (7), show that there is indeed a bend between the Cμ2 and Cμ3 domains, rather than between the Fabs and the IgM-Fc (i.e., not between Cμ1 and Cμ2hen 2). When IgM is bound to antigen, as in the latter structure, the (Cμ2)_2_ domain pair is bent by 100° from its fully extended position with respect to Cμ3 and Cμ4 in the flat disc (7) (**Figure 2A**). Bending of IgM-Fc is an important factor in controlling the complement activity of this antibody. This mechanism is likely preserved through evolution, as jawed fish have multimeric IgM, C1q, and activate complement by the classical pathway (8).

Human IgE-Fc has the same three domain pair composition (Cε2-Cε3-Cε4)_2_ (**Figure 1A**), but the crystal structure revealed an acutely bent conformation with the (Cε2)_2_ domain pair bent back against the Cε3 domains and even making contact with Cε4 (9). This bent structure also predominates in solution (10–13). The angle between the local two-fold axis of the (Cε2)_2_ domain pair and that of the (Cε3-Cε4)_2_ pair was 118° in the crystal structure (8) and this increased even further to 126° when the “high-affinity” receptor for IgE, FcεRI, was bound between the two Cε3 domains, accompanied by an opening up of these domains (14) (**Figure 2Bi**). In contrast, when the “low-affinity” IgE receptor FcεRII/CD23 was bound to a site between the Cε3 and Cε4 domains, the bend decreased by 16° and the Cε3 domains adopted a more closed conformation (15) (**Figure 2Bii**). As a consequence of these opposed conformational changes in the positions of the Cε3 domains relative to each other and to the Cε4 domains, the binding of these two receptors is mutually exclusive—an allosteric effect (16); when one site is accessible, the other is not, and *vice versa*, which is essential for preventing the activation of an allergic reaction *via* FcεRI by multimeric CD23 in the absence of allergen. IgE-Fc can also adopt a fully extended conformation, which can be trapped and stabilised by anti-IgE-Fc antibody Fabs such as the aεFab (17). However, for free IgE-Fc in solution, this extended conformation, to which FcεRI binding is blocked sterically, may be only rarely populated (17). Bending of IgE-Fc is thus important for the modulation of receptor binding activity.

IgY-Fc also has the same homodimeric composition as IgM-Fc and IgE-Fc, (Cυ2-Cυ3-Cυ4)_2_ (**Figure 1A**), but only the crystal structure of the (Cυ3-Cυ4)_2_ domains is known (18) and not that of the (Cυ2)_2_ domain pair, which may be important in receptor and possible complement binding. This is the first structural analysis of the complete IgY-Fc molecule. One receptor, FcRY, is functionally similar to the mammalian transport receptor FcRn and is involved in the transfer of maternal blood IgY to the yolk and then to the embryo (19); it is known to bind to the Cυ4 domains (20) and also to the Cυ3/Cυ4 interface (19). There is also evidence from the modelling of mutated IgY-Fc interactions with FcRY that changes in the angle between the Cυ3 and Cυ4 domains may occur (19), akin to the conformational changes involving the Cε3 domains of IgE-Fc. Intriguingly, the crystal structure of IgY-Fc exhibits different Cυ3-Cυ4 angles in the two chains (18), further indicating that conformational changes involving these domains may be possible. Another IgY receptor was identified in the chicken genome as homologous to mammalian Fc receptors, FcR/L, but the protein has not been prepared (21). A further two receptors are known, ggFcR (22) (on chromosome 20) and CHIR-AB1 (23) (with a gene in the leukocyte receptor complex), which bind to sites homologous to, firstly, the FcεR1 site on IgE-Fc between the Cυ3 domains (22) (**Figure 2Ci**) and secondly, the FcαR1 site on IgA-Fc, close to the CD23-binding site on IgE-Fc between the Cυ3 and Cυ4 domains (24) (**Figure 2Cii**). In chickens, which have only the IgM, IgA, and IgY isotypes, the latter performs the functions of human IgG (25) and IgE (26, 27), but the functions of the two receptors ggFcR and CHIR-AB1, and whether there is any allosteric communication as in IgE, remain to be investigated. Unlike IgM and IgE, the disposition of the (Cυ2)_2_ domain pair of IgY-Fc in solution (or crystal) has not previously been studied.

There is a large gap between the appearance of IgY 310 mya and hominid IgE 2.5 mya, but this is bridged by the monotreme platypus IgE, which appeared 166 mya (28). We have used extant proteins due to their availability and the better, although incomplete, understanding of their function. Our justification for doing so is that the domain structure of all three antibody isotypes has remained constant throughout evolution, and also that for IgM, although its polymeric state has varied, its complement activation function has been conserved.

In this paper, SEC-SAXS (size exclusion chromatography-SAXS) was employed to study the solution conformations of the Fc regions of these four antibodies. In the case of IgM-Fc, the homodimeric subunit of the human pentamer or hexamer was used to permit comparison with the other antibody Fc domains (**Figure 1**). SEC-SAXS measures the X-ray scattering as the protein is eluted from a size exclusion column, to better resolve monomeric from aggregated material. The scattering data not only permit analysis of the average conformation of each protein in solution but also detect whether more than one, i.e., an ensemble, of different conformations is present. We tracked the evolution of conformational variability and function through this series of antibodies.

## Materials and methods

### Protein preparation

All four Fc proteins, human IgE-Fc (huIgE-Fc), platypus IgE-Fc (plIgE-Fc), chicken IgY-Fc (chIgY-Fc), and human IgM-Fc (huIgM-Fc), are glycosylated. huIgE-Fc has three N-glycosylation sites at asparagine residues 265, 371, and 397. The latter residue plays a structural role in the Fc, located “internally” between the two Cε3 domains, and was retained, whereas N265 and N371 were mutated to glutamine. In plIgE-Fc, chIgY-Fc, and huIgM-Fc, the homologous site to 397 was retained, but other sites identified in biochemical studies or using Net-Gly (29) were mutated to glutamine.

huIgE-Fc (N265Q, N371Q) was secreted from a stable NS-0 cell line and purified from tissue culture supernatant by cation exchange chromatography (17). The supernatant was buffer-exchanged into 50 mM sodium acetate (pH 6.0) and 75 mM NaCl and loaded onto an SPHP cation-exchange column (GE Healthcare). huIgE-Fc (N265Q, N371Q) was eluted with a 10 × column volume gradient into 50 mM sodium acetate (pH 6.0) and 1 M NaCl. The eluted fractions were pooled, concentrated, and further purified by SEC on a Superdex G200 column (GE Healthcare) in PBS (pH 7.4).

The protein sequences of the other three Fc fragments, chosen to align with the N and C termini of the huIgE-Fc, are shown in **Supplementary Table 1**. C414 in huIgM-Fc, which mediates pentamer or hexamer formation, was mutated to serine. Sequences were pipe-cloned (30) into pcDNA5, preceded by a mouse kappa light chain leader sequence, and followed by bases coding for six histidine residues to facilitate secretion of the Fc proteins by expression in HEK293F cells and purification, respectively. Cell supernatants were purified on a Ni-NTA column (Thermo Fisher Scientific) followed by SEC chromatography in Tris-buffered saline with azide (pH 7.5) using a Superdex G200 column (GE Healthcare).

Protein purity was assessed using reduced 5 μg aliquots on a 10% SDS-PAGE gel, and the molecular mass was estimated by size exclusion chromatography-multi-angle laser light scattering (SEC-MALLS), which combines multi-angle light scattering with size-exclusion chromatography to estimate a shape-independent molecular mass. Glycosylation at the conserved N-glycosylation site was checked through comparison of the molecular mass of 2.5 μg of protein samples incubated with or without PNGaseF (NEB, P0704S) according to the manufacturer’s instructions. The gel was calibrated with molecular mass markers by plotting log molecular mass vs. migration (31).

### SAXS data collection

Conditions for data collection at the Diamond Light Source (BL21) are shown in **Supplementary Table 2**. Each of the four proteins was prepared twice. Data were collected for the first set of four proteins by SEC-SAXS with a Shodex KW403 column and for the second set with a Superdex G200 Increase column. The Rg values for each set, i.e., the two experimental replicates for each protein, are shown in **Supplementary Table 3**.

### FPMod models

The conformational space occupied by all four proteins was sampled using models of huIgE-Fc and huIgM-Fc generated by FPMod (32). Models for plIgE-Fc and huIgE-Fc were generated from the acutely bent (9) (PDB 1O0V) and fully extended (17) (PDB 4J4P) crystal structures of huIgE-Fc, arranged as two rigid bodies (Cε2)_2_ and (Cε3-Cε4)_2_, joined in one of the chains by the flexible linker of three amino acids, DSN. Models for huIgM-Fc and chIgY-Fc were generated from a model of fully extended huIgM-Fc (using the sequence of huIgM-Fc with the crystal structure of fully extended huIgE-Fc as a template), again arranged as two rigid bodies and using a flexible linker of seven amino acids, VPDQDTA (from IgM-Fc), in one of the chains. These seven residues are not visible in the cryo-EM structure of full-length human IgM (6) (PDB 8ADY), and therefore they are probably flexible. The preceding cysteine forms a disulphide bond between the heavy chains (33, 34).

### Ensemble selection

The intensity plots from the first set of protein preparations were used as input to a non-negative linear least-squares algorithm, NNLSJOE^1^ (35, 36), in EOM 3.0. The pool of models (of huIgE-Fc for the huIgE-Fc and plIgE-Fc intensity plots and of huIgM-Fc for the huIgM-Fc and chIgY-Fc intensity plots) was analysed by NNLSJOE, resulting in a set of several models for each protein that best fit the corresponding experimental intensity plot. The number of runs (35) was 100. Fits with values of χ^2^<2 were accepted. Each set of models was distributed according to Rg values into four bins representing acutely bent, partially bent, and fully extended protein conformations.

## Results

### Protein preparation

**Supplementary Figure 1A** shows the purity of the proteins as determined by SDS PAGE; the molecular masses, estimated by SEC-MALLS, of huIgM-Fc, chIgY-Fc, plIgE-Fc, and huIgE-Fc were 72, 79, 80, and 73 kD, respectively. Reduced heavy chains of each protein can be seen with a molecular mass of just >35kD, consistent with their calculated sizes of 36 to 40 kD. Evidence for glycosylation at the conserved N-glycosylation site can be seen in **Supplementary Figure 1B**, which compares each of the four proteins by SDS-PAGE, incubated with (+) or without (-) PNGaseF. These each show a molecular mass difference of several kD; the crystal structures of huIgE-Fc (PDB 1O0V and PDB 2WQR) have approximately 2 kD of carbohydrate on both chains together (9, 14).

### SAXS analysis

SAXS analysis permits calculation of the radius of gyration, Rg, a measure of the distance distribution of mass about the centre of gravity of the protein; more extended conformations have larger Rg values than compact structures. The full X-ray scattering curves (intensity as a function of scattering angle) are sensitive to details of protein shape, and as described in the following section, these were analysed using a non-negative linear least-squares algorithm to choose from a pool of models those that best fit the data and determine whether one or a number of different conformations was present in solution.

**Figure 3:**
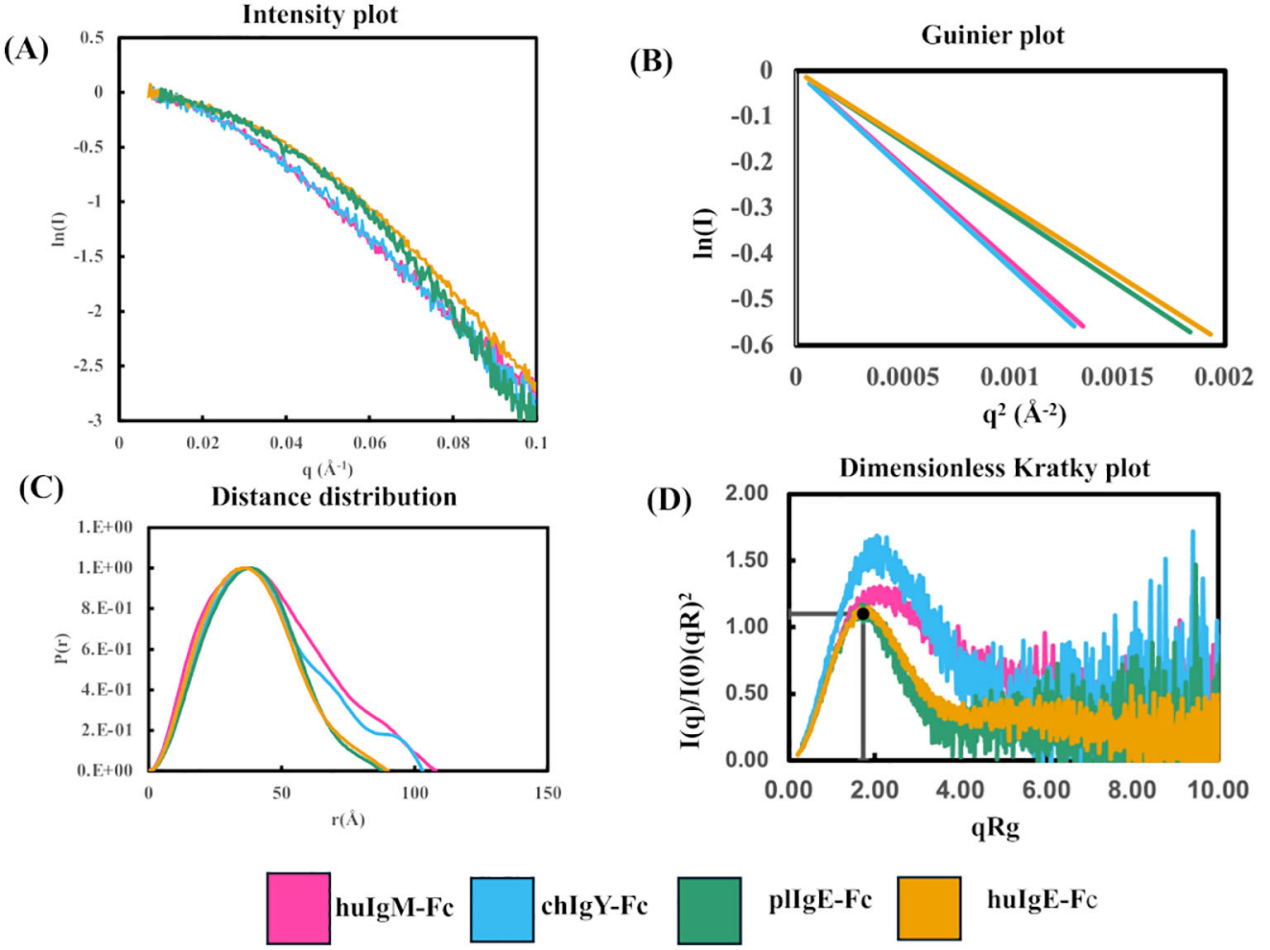
Comparison of SAXS analyses of the four recombinant Fc proteins (Preparation 1) using Scåtter. **(A)** Intensity plot. **(B)** Guinier plot. **(C)** P(r) plot. **(D)** Dimensionless Kratky plot. In panels **(A, B)** the first point on each plot has been transposed to (0,0) to facilitate a comparison of the Rg values for the four proteins huIgM-Fc (pink), chIgY-Fc (blue), plIgE-Fc (green), and huIgE-Fc (yellow).

**Figure 4:**
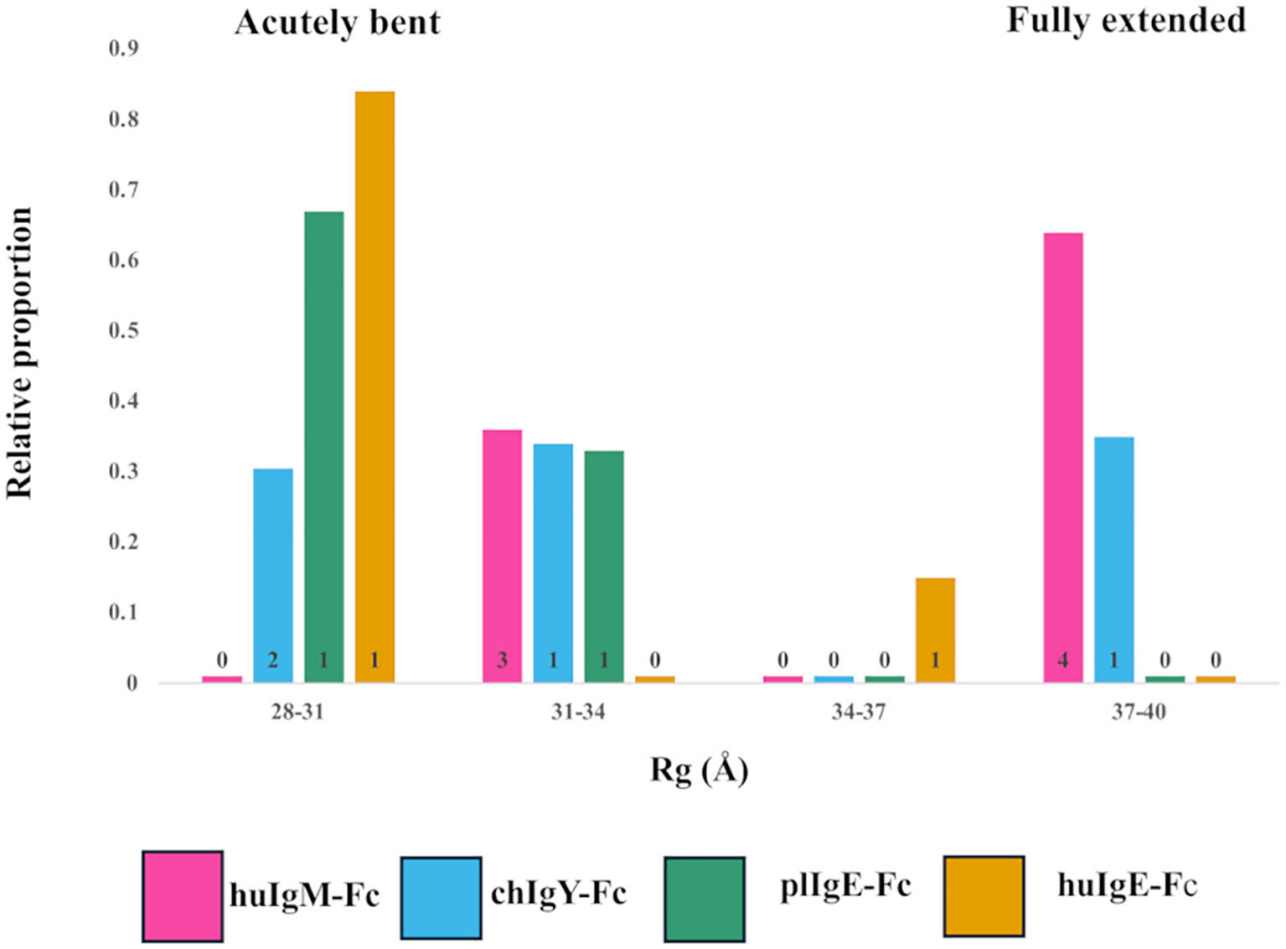
Models selected to best fit the X-ray scattering data for the four recombinant Fc proteins (Preparation 1). Relative contributions and binned Rg values for each of the models selected for human huIgM-Fc (pink), chIgY-Fc (blue), plIgE-Fc (green), and huIgE-Fc (yellow) are shown. Four bins were chosen to cover the range of Rg values from acutely bent (28-31Å), to partially bent (31-34Å and 34-37Å) to fully extended (37-40Å). The number of models contributing to each bar is indicated.

Two separate sets of preparations of the four Fc proteins were used for the collection of SAXS data and the calculation of Rg values. **Supplementary Table 3** shows that the Rg values are similar for the two sets of preparations. **Supplementary Figure 2**, and **Figures 3** and **4** report data from the first preparation.

The data were imported into Chromixs (37) in ATSAS 2.8 (38) for buffer subtraction and the generation of elution profiles (**Supplementary Figure 2**), and into Scåtter (39) for the subsequent generation of intensity, Guinier, P(r), and dimensionless Kratky plots (**Figure 3**). Some aggregation was evident in huIgM-Fc, chIgY-Fc, and plIgE-Fc (**Supplementary Figures 2A–C**). Consequently, the frames 253-262, 270-278, 248-258, and 278-291 for the four proteins huIgM-Fc, chIgY-Fc, plIgE-Fc, and huIgE-Fc, respectively, were used to generate intensity plots. Calculated average Rg values for each of the frames (1 s exposure) were used to assess the range of average Rg values across the part of the peak used (**Table 1**).

**Table 1:** Stability of Rg values across the elution profile frames in preparation 1 used for analysis.

	huIgM-Fc	chIgY-Fc	plIgE-Fc	huIgE-Fc
Average calculated Rg (Å)	36.8	35.6	30.4	29.7
Range of average Rg values across frames used	2.59	2.63	0.84	0.59

Elution profiles are shown in **Supplementary Figure 2**.

**Figure 3A** shows the intensity plots for each of the four proteins. Average Rg values in reciprocal space were estimated using a Guinier plot (ln I vs. q^2^ gives a straight line with slope –Rg/3) (**Figure 3B**) with qRg values below 1.3 and residuals symmetric about zero (40). The lower average Rg values for huIgE-Fc and plIgE-Fc (29.7Å and 30.4Å), compared with chIgY-Fc and huIgM-Fc (35.6Å and 36.8Å), indicate that the IgE-Fc molecules adopt a more compact structure on average than chIgY-Fc and huIgM-Fc, which adopt, on average, more extended structures (**Table 1**). **Figure 3A**, showing only the intensity plot below q = 0.1 Å^-1^, emphasises this difference between the more extended (huIgM-Fc and chIgY-Fc) and more compact (plIgE-Fc and huIgE-Fc) configurations at q ~ 0.05 Å^-1^.

It is striking that the range of average Rg values over the frames used for each protein analysis is significantly smaller for plIgE-Fc and huIgE-Fc than for huIgM-Fc and chIgY-Fc (**Table 1**). This most likely reflects the conclusion reached from the analysis presented in the following section, that both IgE-Fc molecules predominantly adopt an acutely bent compact conformation and thus represent a more structurally homogeneous population of molecules than chIgY-Fc and huIgM-Fc, which adopt a greater range of conformations from bent to extended. These different conformations may be differentially retarded on the column, and consequently, the average Rg values vary to a greater extent across the peak for chIgY-Fc and huIgM-Fc. These ranges of average Rg values for each protein derived from the first preparation (**Table 1**) also encompass the range of values derived from the second preparation (**Supplementary Table 3**). Although we cannot place statistical significance estimates on these Rg values, given only two experimental replicates, those Rg values derived from the second preparation add confidence to the values and ranges reported in **Table 1**.

The maximum dimension of each protein, D_max_, was estimated from the P(r) function (**Figure 3C**). This shows that the maximum lengths of huIgM-Fc and chIgY-Fc (approximately 110Å and 105Å, respectively) are greater than those of plIgE-Fc and huIgE-Fc (approximately 90Å). (Values for Preparation 2 are in good agreement; huIgM-Fc, ~116Å; chIgY-Fc, ~104Å; plIgE-Fc, ~94Å; and huIgE-Fc, ~98Å.) A dimensionless Kratky plot [where (qRg)^2^ I(q)/I(0) is plotted against qRg] was used to assess the globularity of the proteins (**Figure 3D**). huIgE-Fc and plIgE-Fc appear globular with a characteristic maximum close to y = 1.1, x = √3, whereas huIgM-Fc and chIgY-Fc have a peak at higher x values, suggesting a more extended protein (40).

Although the experimental Rg values discussed above are average values for each protein, it is instructive to compare them with the Rg values calculated for two crystal structures of huIgE-Fc discussed earlier in the Introduction. One of these is free huIgE-Fc (9) (PDB 1O0V), which is acutely bent between the Cε2 and Cε3 domains and adopts a very compact structure; the other is that of huIgE-Fc stabilised in a complex in a fully extended conformation (17) (PDB 4J4P). The calculated Rg value for the former is 28.8Å, and for the latter, 35.6Å (**Supplementary Table 4**). The experimental average Rg values of huIgE-Fc (29.7Å) and plIgE-Fc (30.4Å) are close to the value for the acutely bent huIgE-Fc structure. In contrast, the experimental average Rg values of huIgM-Fc (36.8Å) and chIgY-Fc (35.6Å) are close to the value for the fully extended huIgE-Fc structure. This is also true for the average Rg values derived from the second protein preparation (**Supplementary Table 3**).

### Models for interpretation of the SAXS data

Ensembles of models were generated, which maximise the fit of the theoretical to the experimental intensity plots, using a pool of either huIgE-Fc models or huIgM-Fc models (see Materials and Methods). The ensembles chosen by NNLSJOE consisted of a minimum of two and a maximum of seven models and thus always included structures other than the predominant acutely bent or fully extended configuration. **Figure 4** shows the Rg values of each of the models chosen and their relative contribution the fit of the scattering data for each of the four proteins.

For huIgM-Fc, the SAXS results show that no acutely bent structures are present. There are, however, clusters of both fully extended and partially bent structures (**Figure 4**). The former constitute 64% of the structures [four models with Rg values of 38.0Å (15%), 38.1Å (27%), 38.2Å (19%), and 38.5Å (3%)], and the latter 36% of the structures [three models with Rg values of 32.8Å (14%), 32.8Å (6%), and 33.3Å (16%)]. Since bending of the IgM-Fc is proposed to expose the binding site for C1q (4, 5), the question arises: which of the conformations observed are capable of binding C1q? Complement binding residues in IgM-Fc have been identified by site-directed mutagenesis (41), and they are inaccessible to C1q in the fully extended IgM-Fc molecule. However, the complex of C1q bound to the homologous region of human IgG-Fc in the cryo-EM model (42) (PDB 6FCZ) can be used to assess whether C1q might bind to the partially bent structures of huIgM-Fc observed in the present study. The (Cγ2-Cγ3)_2_ domains of IgG-Fc in the C1q complex (PDB 6FCZ) were superposed on each of the seven models of huIgM-Fc selected by the algorithm NNLSJOE, using matchmaker from ChimeraX, and in the four fully extended models representing 64% of the material, the Cμ2 domains clashed sterically with the C1q “head”. The other three, partially bent models, did not clash. Two of these superpositions, one clashing and one not, are shown in **Supplementary Figure 3**. The fact that this implies that 36% of the material adopts conformations sufficiently bent to allow access to C1q is discussed below.

Although for huIgM-Fc there are no acutely bent models represented, chIgY-Fc contains material that is acutely bent, partially bent, and fully extended (**Figure 4**; **Supplementary Figure 4**). These constitute 31%, 34%, and 35% of the structures, respectively (Rg values for each of the models are shown in **Supplementary Figure 4**). In contrast, plIgE and huIgE are predominantly acutely bent, although both indicate the presence of some partially bent material (**Figure 4**). For plIgE-Fc, the acutely bent structure constitutes 67% of the material (Rg 28.7Å), with 33% partially bent (Rg 31.4Å), and for huIgE-Fc, 84% (Rg 29.2Å) is acutely bent and 16% (Rg 35Å) is partially bent. Neither plIgE-Fc nor huIgE-Fc show evidence of any fully extended material. The four proteins form a series in which the proportion of acutely bent material increases: IgM-Fc < IgY-Fc < plIgE-Fc < huIgE-Fc. This follows their order of appearance in evolution. The ensemble of conformations for IgY-Fc from acutely bent to fully extended (**Figure 4**; **Supplementary Figure 4**), rather than the predominantly acutely bent conformation of IgE-Fc and predominantly fully extended conformation of IgM-Fc, reflects IgY’s position as an evolutionary intermediate between IgM and IgE.

These conclusions are supported by analysis using NNLSJOE of the data from preparation 2. Although there are some differences in the proportions of partially bent conformations, there is very good agreement for the proportions of acutely bent and fully extended conformations. The proportion of acutely bent material increases IgM-Fc < IgY-Fc < plIgE-Fc < huIgE-Fc (0%, 31%, 77%, and 83% for preparation 2, *cf.* 0%, 31%, 67%, and 84% for preparation 1); huIgM-Fc displays no acutely bent conformations and is predominantly fully extended (70% for preparation 2, *cf.* 64% for preparation 1); huIgE-Fc and plgE-Fc are predominantly acutely bent (83% and 77%, respectively, for preparation 2, *cf.* 84% and 67% for preparation 1) and display no fully extended conformations; and chIgY-Fc displays both acutely bent and fully extended conformations (31% and 69%, respectively, for preparation 2, *cf.* 31% and 35% for preparation 1).

## Discussion

Homodimeric Fc regions (C_H_2-C_H_3-C_H_4)_2_ of the four antibodies, human IgM, human and platypus IgE, and chicken IgY, were prepared with glycosylation at the conserved “internal” site in C_H_3, for analysis of their solution conformation. SEC-SAXS was employed, and the intensity data were analysed using a non-negative linear least-squares algorithm to choose from a pool of models to fit the data and determine whether one or a number of different conformations were present in the solution. The analysis suggests the presence of more than one conformation in each case.

The crystal structure of huIgE-Fc reveals an acutely bent conformation (9), which appears also to predominate in solution, as determined by earlier SAXS (11, 13) and FRET (10, 12) analyses. The energy landscape for IgE-Fc has been explored by molecular dynamics simulations, and an estimate of the energy difference between the acutely bent and fully extended conformations (approximately 20 kJ/mol) suggests that there is little extended IgE-Fc in solution^17.^ This was confirmed in the present study, with 84% of the material estimated to be in an acutely bent conformation (**Figure 4**).

In contrast, for huIgM-Fc, the SAXS results show that no acutely bent structures are present, but there are both fully extended and partially bent structures (**Figure 4**). We have shown, by comparison with the cryo-EM model of the C1q “head” bound to the homologous IgG-Fc (PDB 6FCZ) as described above (**Supplementary Figure 3**), that in 36% of these structures, the C1q binding site is accessible. However, free pentameric IgM does not bind C1q or activate complement, suggesting that the formation of the pentamer (or hexamer) may affect the conformational diversity in IgM-Fc. Although pentameric IgM (6) and IgM-Fc (43–45) cryo-EM structures imply that flexibility and bending at the Cμ2-Cμ3 interface can occur, even in the absence of antigens, the extent of this bending is not known. Other factors, such as glycosylation, may also affect the ability of IgM-Fc to bend and activate complement. A recent study showed that in the sera of patients with a severe case of COVID-19, increased glycosylation of IgM-Fc correlated with increased IgM-dependent complement deposition (46). We note that in the recently determined structures of the IgM B-cell receptor (33, 34, 47), the Fc region adopts an extended conformation, perhaps stabilised by the accessory Igα and Igβ molecules.

IgE-Fc from platypus, the most evolutionarily primitive of the extant mammals, was included in this study to offer an evolutionary intermediate between chicken IgY-Fc and human IgE-Fc. In fact, the profile of conformations seen for plIgE-Fc is very similar to that of huIgE-Fc, dominated by an acutely bent conformation (67%, **Figure 4**). CD23 has been annotated in the platypus genome (28), and fragments of an FcεR1α homologue have been identified with Blast (48). However, key residues in the huIgE-Fc-binding site for CD23 (15, 16) are not conserved in plIgE-Fc, and binding of platypus FcεR1 to plIgE-Fc has never been demonstrated, nor have there been reports of an allergic reaction, although one individual of the only other extant monotreme, the echidna, was found to be allergic to its main source of food, namely ants (49). The presence of IgE, CD23, and possibly FcεR1α in the platypus genome suggests that the system seen in humans (50) had already evolved to combat helminths that infect monotremes and marsupials (51).

Acutely bent and fully extended structures occur in the ensemble for chIgY-Fc, together with intermediate conformations, which are clearly different from the distributions seen for either IgM-Fc or IgE-Fc (**Figure 4**). The classical complement pathway has been found in chicken (52), but its activation could be due to IgY or IgM. There are no structural or mutational data to indicate how chicken IgY might interact with chicken complement, but two out of the three important residues in both the human IgG and IgM interaction with human complement, P329 and P331, align with prolines in IgY. Chicken IgY only exists as a homodimer (in contrast to the pentameric or hexameric IgM), and the SAXS results show that 31% of it [as determined by performing the same superposition of the IgG-Fc/C1q complex (42) (PDB 6FCZ) as described above for IgM-Fc] is bent sufficiently to expose the complement binding site if it exists; the disposition of the (Cυ2)_2_ pair is, therefore, unlikely to affect activation. The range of conformations for chicken IgY-Fc and the putative C1q-binding site is illustrated in **Supplementary Figure 4**. If IgY can activate chicken complement, the formation of polymeric structures upon antigen binding is likely to be the control mechanism, as it is with IgG (53, 54), because enough of the IgY-Fc is sufficiently bent to allow C1q to bind. Although chickens do experience anaphylaxis (26), there is no evidence that IgY and a receptor are involved. On the other hand, it is known that IgY has some functions analogous to those of both human IgG and IgE, which may involve the identified receptors ggFcR (22) and CHIR-AB1 (23), which bind to IgY-Fc at similar locations, respectively, to FcεRI and CD23 on IgE-Fc (22, 24) (**Figures 2B, C**). The presence of more than one bent conformation suggests that, like huIgE-Fc, different dispositions of the (Cυ2)_2_ domain pair may correspond to the binding of different chicken receptors, and changes in the Cυ3-Cυ4 angle may also occur (18, 19), as described in the Introduction, similar to those that are associated with the open and closed conformations of the Cε3 domains in IgE-Fc (14, 15). Allostery in relation to IgY receptor binding might therefore be possible, as it is for IgE.

We have studied and compared the solution structures of human IgM-Fc, platypus and human IgE-Fc, and chicken IgY-Fc, the latter being the first structural study of the complete Fc region of this isotype. While IgM-Fc is principally fully extended in solution, with no acutely bent conformations, IgE-Fc is principally acutely bent in solution, with no fully extended conformations. In contrast to both IgM-Fc and IgE-Fc, IgY-Fc displays an ensemble of conformations ranging from acutely bent to fully extended, reflecting IgY’s position as an evolutionary intermediate between IgM and IgE. Indeed, the proportion of acutely bent conformation, IgM-Fc < IgY-Fc < plIgE-Fc < huIgE-Fc, follows their order of appearance in evolution. The solution structure of human IgM-Fc (which was already present 425 mya in jawed fish) is consistent with IgM using the bend between the (Cμ2)_2_ domain pair and (Cμ3-Cμ4)_2_, influenced by glycosylation and/or multimerisation, to control complement activation upon antigen binding. Hominid IgE (which only appeared 2.5 mya) does not bind complement, but the bend between (Cε2)_2_ and (Cε3-Cε4)_2_ is associated with the mutually exclusive allosteric binding of two receptors, FcεRI and CD23. This function probably also exists in the monotremes platypus and echidna (which first appeared 166 mya). The solution structure of IgY-Fc (which appeared 310 mya in tetrapods) suggests that it has lost the ability to control complement binding using the (Cυ2)_2_ domain pair and may have already co-opted that binding site for Fc receptor binding, opening up the possibility of allosteric control of receptor binding as seen in human IgE.

In summary, IgY occupies an intermediate position in the evolution of antibody structure from the most primitive IgM to the most recent IgE. This is the first structural analysis of the complete IgY-Fc that allows direct comparison with IgM-Fc and IgE-Fc; we found that IgY-Fc displays a unique ensemble of conformations in solution, distinct from IgM-Fc and IgE-Fc but displaying features of both. We anticipate that this unique structure of IgY-Fc will be reflected in unique biology and function, in particular with respect to its receptor interactions, further illustrating the co-evolution of antibody structure and function.

## Data Availability

The original contributions presented in the study are publicly available. This data can be found in the Dryad repository here: https://datadryad.org/stash/dataset/doi:10.5061/dryad.6q573n66m.

## Footnotes

1
https://www.embl-hamburg.de/biosaxs/manuals/eom.html

## Supplementary material

Supplementary Figure 1
**(A)** SDS-PAGE analysis under reducing conditions of all four recombinant antibody Fc proteins; tracks for plIgE-Fc and chIgY-Fc are duplicated. **(B)** SDS-PAGE analysis under reducing conditions of all four antibody Fc proteins in the presence (+) and absence (-) of PNGaseF, showing evidence of glycosylation.

Supplementary Figure 2 SEC-SAXS elution profiles for **(A)** huIgM-Fc, **(B)** chIgY-Fc, **(C)** plIgE-Fc, and **(D)** huIgE-Fc generated using Chromixs. The frames for analysis, respectively 253-262, 270-278, 248-258, and 278-291, indicated by the bars, were chosen to minimise aggregation, as described in the text.

Supplementary Figure 3 Two of the IgM-Fc models (shown in pink), one partially bent **(A)** and one fully extended **(B)**, chosen to fit the SAXS intensity plot for this protein, are each superimposed on the structure of the complex (shown in yellow) of IgG-Fc bound to the “head” domain of C1q (PDB:6FCZ). The C1q head domain (yellow) is seen clearly at the top of panel A, and the IgG-Fc domains (Cγ2 and Cγ3, also in yellow) are superimposed on the homologous IgM-Fc domains (Cμ3 and Cμ4, shown in pink). The Cμ2 domains of IgM-Fc (pink) can be seen bent to the left in panel **(A)** and extended upwards in panel **(B)** Panels **(A, B)** are shown in the same orientation. In panel **(A)** there is no clash between the Cμ2 domains (or any other part) of IgM-Fc (pink) and the head domain of C1q (yellow), and thus C1q is expected to bind to this bent conformation of IgM-Fc. Two key residues in the C1q-binding site on IgM-Fc, Pro329, and Pro331 (refer to text), are indicated as black spheres, two on each Cμ3 domain. In panel **(B)**, the Cμ2 domains of IgM-Fc (pink) in the fully extended conformation clash with the head domain of C1q (yellow), and thus C1q cannot bind to this conformation of IgM-Fc.

Supplementary Figure 4 The four models **(A–D)** selected to fit the SAXS intensity plot for IgY-Fc, shown in order of their increasing Rg values (28.7Å, 30.2Å, 33.7Å, and 38.5Å), from acutely bent to fully extended. The Cυ2 domain pairs are coloured dark blue to highlight the bending. The fractional contribution of each conformation to the ensemble (respectively 6%, 25%, 34%, and 35%) results in an average Rg value of 35.1Å, which is very close to the average Rg value of 35.6Å reported in **Table 1**. Two key proline residues in the C1q-binding site of IgM (see **Supplementary Figure 3** and refer to the text) are conserved in Ig, and are indicated here (black spheres on the Cυ3 domain at the front, grey spheres on the Cυ3 domain at the back). If C1q binding to IgY does occur, we expect the site to be accessible in conformations **(A, B)**, but not **(C, D)**.

Supplementary Table 1 Protein sequences, with His-tags, of human IgM-Fc, chicken IgY-Fc, and platypus IgE-Fc, aligned with the human IgE-Fc sequence (ac. no. P01854) to span from the N to C termini of the fragment described in ref. 17.

Supplementary Table 2 SAXS data collection parameters.

Supplementary Table 3 Rg values for two preparations of each protein were estimated from the SAXS intensity plot and the Guinier equation in Scåtter. A Shodex KW403 column was used for preparation 1 and a Superdex G200 Increase for preparation 2 (see Materials and Methods).

Supplementary Table 4 Rg values calculated from crystal structures of acutely bent (PDB 1O0V) and fully extended (PDB 4J4P, without Fabs) huIgE-Fc, and minimum and maximum Rg values for models of huIgM-Fc and huIgE-Fc, calculated with CRYSOL (1).
