# Supplementary material for: The evolution of flexibility and function in the Fc domains of IgM, IgY, and IgE
Source: Front Immunol. 2024 Oct 9;15:1389494. doi: 10.3389/fimmu.2024.1389494 (PMC11496790; doi:10.3389/fimmu.2024.1389494)
Supplement: Supplementary Figure 1 — (A) SDS-PAGE analysis under reducing conditions of all four recombinant antibody Fc proteins; tracks for plIgE-Fc and chIgY-Fc are duplicated. (B) SDS-PAGE analysis under reducing conditions of all four antibody Fc proteins in the presence (+) and absence (-) of PNGaseF, showing evidence of glycosylation. [file DataSheet1.pdf]

The evolution of flexibility and function in the Fc domains of IgM, IgY and IgE

Rosaleen A. Calvert[1*], Rosemary A. Nyamboya[1], Andrew J. Beavil[1] and Brian J. Sutton[1*]

[1]Randall Centre for Cell and Molecular Biophysics, Faculty of Life Sciences and Medicine, King's College London, SE1 1UL
[*]Corresponding authors: rosy.calvert@kcl.ac.uk; brian.sutton@kcl.ac.uk

Supplementary information

Supplementary Table 1. Protein sequences, with His-tags, of human IgM-Fc, chicken IgY-Fc and platypus IgE-Fc, aligned with human IgE-Fc sequence (ac. no. P01854) to span from the N- to C-termini of the fragment described in ref. 17.

huIgM-Fc (ac. no. P01871)<br>DILPVIAELPPKVSVFVPPRDGFFGNPRKSKLICQATGFSPRQIQVSWLREGKQVGSGVTT<br>DQVQAEAKESGPTTYKVTSTLTIKESDWLSQSMFTCRVDHRGLTFQQQASSMCVPDQDTAI<br>RVFAIPPSFASIFLTKSTKLTCLVTDLTTYDSVTISWTRQNGEAVKTHTQISESHPNATFS<br>AVGEASISEDDWNSGERFTCTVTHTDLPSPLKQTISRPKGVALHRPDVYLLPPAREQLNLR<br>ESATITCLVTGFSPADVFVQWMQRGQPLSPEKYVTSAPMPEPQAPGRYFAHSILTVSEEEW<br>NTGETYTCVVAHEALPNRVTERTVDKSTGKHHHHHH
chIgY-Fc (ac. no. S00390)<br>DIVARVGPPLPVPPEVQVLHASSCTPSQSESVELLCLVTGFSPASAEVEWLVDGVGGLLVA<br>SQSPAVRSGSTYSLSSRVQVSGTDWREGKSYSCRVRHPATNTVVEDHVKGCPDGAQSCSPI<br>QLYAIPPSPGELYISLDAKLRCLVVNLPSDSSLSVTWTREKSGNLRPDPMVLQEHFNGTYS<br>ASSAVPVSTQDWLSGERFTCTVQHEELPLPLSKSVYRNTGPTTPPLIYPFAPHPEELSLSR<br>VTLSCLVRGFRPRDIEIRWLRDHRAVPATEFVTTAVLPEERTANGAGGDGDTFFVYSKMSV<br>ETAKWNGGTVFACMAVHEALPMRFSQRTLQKQAGKHHHHHH
plIgE-Fc (ac. no. AAL17702)<br>DIDASKDPIPPTVKLLHSSCDPRGDSQASIELLCLITGYSPAGIQVDWLVDGQKAENLFPY<br>TAPPKREGQRSFSSHSEVQITQDQWLSGKTFTCQVTHLADKKTYQDSARKCADSDPRGITV<br>FLTPPSPTDLYISKTPKLTCLIIDLVSTEGMEVTWSRESGTPLSAESFEEQKQFNGTMSFI<br>STVPVNIQDWNEGESYTCRVAHPDLPSPIIKTVTKLPGKRLAPEVYAFPPHQAEVSHGDSL<br>SLTCLIRGFYPEQISVRWLLDNKPLPTEHYRTTKPLKDQGPDPAYFLYSRLAVNKSTWEQG<br>NVYTCQVVHEALPSRNTERKFQHTSGNHHHHHH

Supplementary Table 2. SAXS data collection parameters.

Instrument	Diamond Light Source, Beamline B21
Beam size at sample	250 x 250 μm
Wavelength	0.54-2.07 Å
Q range	0.002-0.42 Å
Detector	Pilatus 2M
Detector distance	4.014 m
Exposure per image	1 sec at 3 sec intervals
Column	Shodex KW403 or Superdex G200 Increase
Flow rate	0.15 or 0.3 mL/min
Sample concentration	5.00 mg/mL
Sample size	50 μL
Buffer	0.15 M NaCl, 50 mM Tris, 0.1% sodium azide pH 7.5
Temperature	20°C

Supplementary Table 3. Rg values for two preparations of each protein estimated from the SAXS intensity plot and Guinier equation in Scåtter. A Shodex KW403 column was used for preparation 1 and a Superdex G200 Increase for preparation 2 (see Materials and Methods).

Prep.	Reciprocal/real space values of Rg (Å) from Scåtter			
	huIgM-Fc	chIgY-Fc	plIgE-Fc	huIgE-Fc
1	36.48/35.54	35.72/34.59	30.38/30.70	29.78/29.60
2	37.24/37.20	37.25/37.42	30.41/30.25	28.94/28.91

Supplementary Table 4. Rg values calculated from crystal structures of acutely bent (PDB 1O0V) and fully extended (PDB 4J4P, without Fabs) huIgE-Fc, and minimum and maximum Rg values for models of huIgM-Fc and huIgE-Fc, calculated with CRYSOL[1].

Rg (Å) from PDB crystal structures and models (min. and max.) calculated by CRYSOL[1]			
PDB 1O0V	PDB 4J4P	huIgM-Fc models	huIgE-Fc models
28.83	35.59	29.00 min	28.56 min
		39.53 max	37.29 max

Supplementary Figure 1.

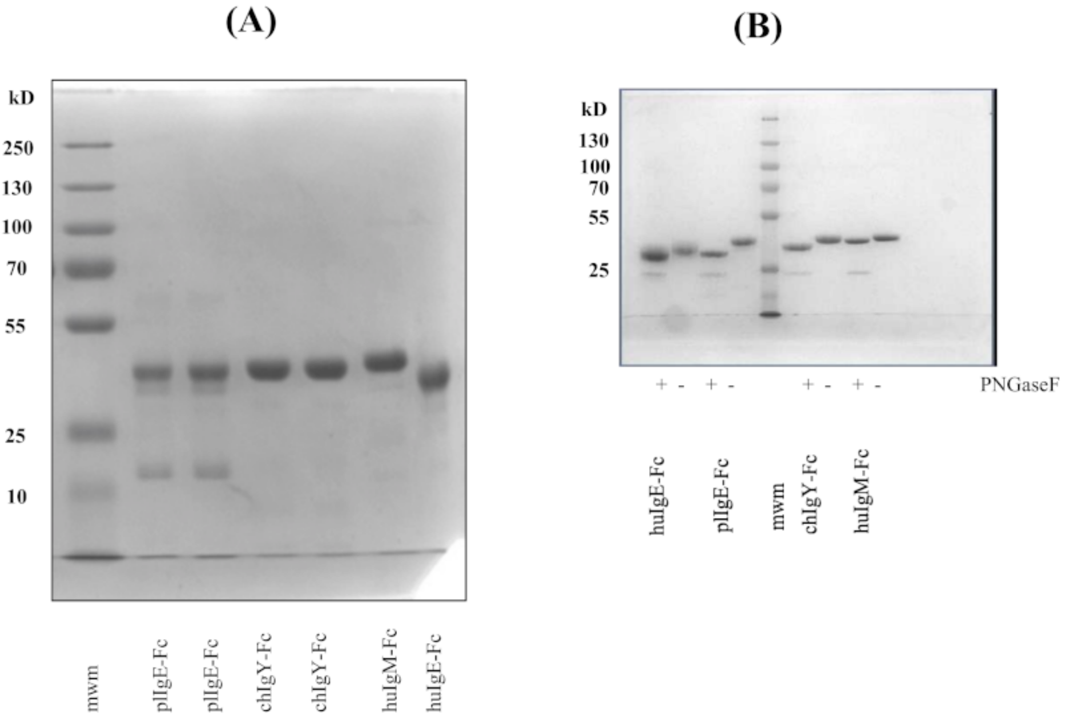

Supplementary Figure 1

(A) SDS-PAGE analysis under reducing conditions of all four antibody Fc recombinant proteins; tracks for plIgE-Fc and chIgY-Fc are duplicated. **(B)** SDS-PAGE analysis under reducing conditions of all four antibody Fc proteins in the presence (+) and absence (-) of PNGaseF, showing evidence of glycosylation.

Supplementary Figure 2.

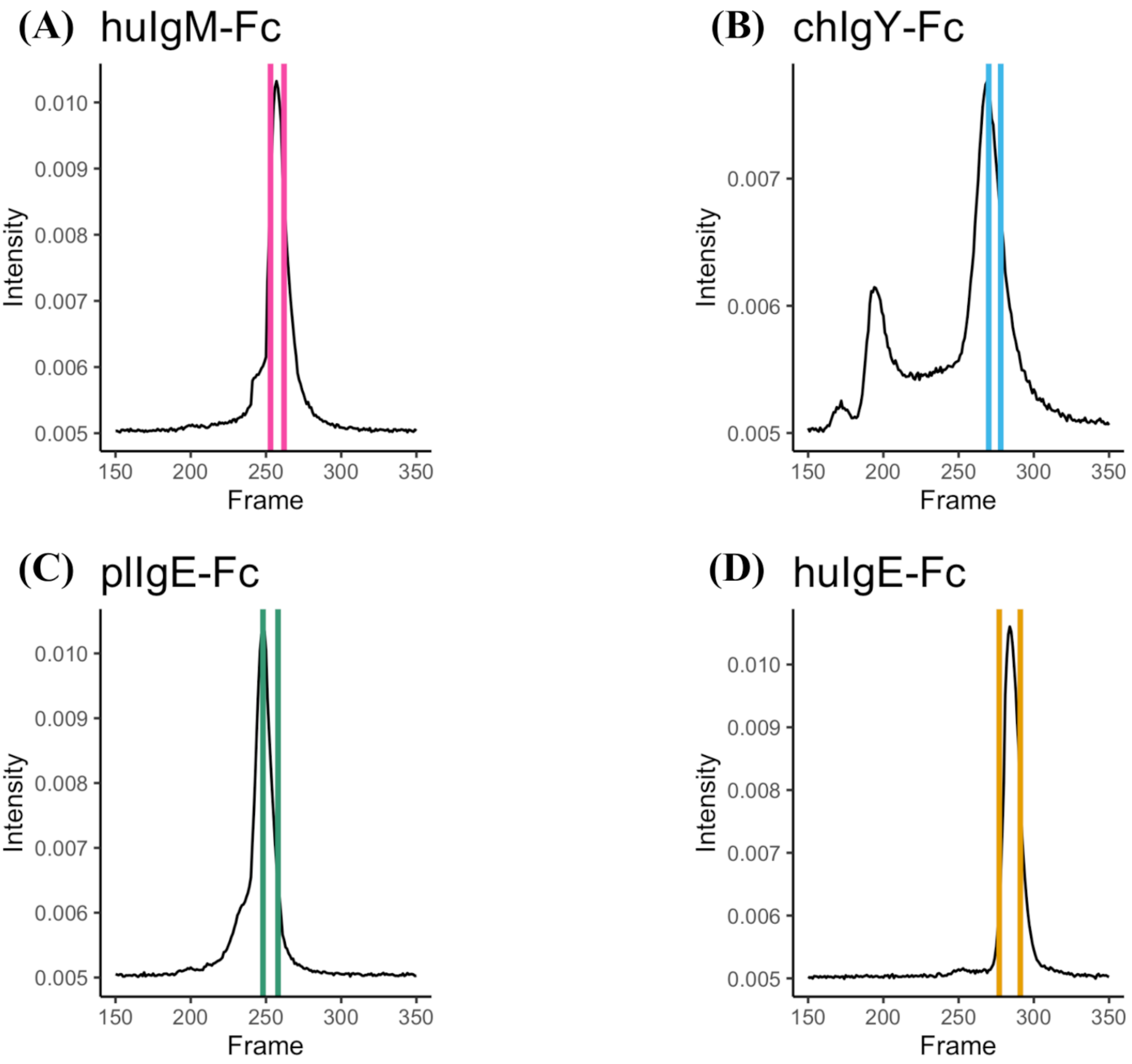

Supplementary Figure 2.
SEC-SAXS elution profiles for **(A)** huIgM-Fc; **(B)** chIgY-Fc; **(C)** plIgE-Fc; **(D)** huIgE-Fc generated using Chromixs. Frames for analysis, respectively 253-262, 270-278, 248-258, and 278-291, indicated by the bars, were chosen to minimise aggregation, as described in the text.

Supplementary Figure 3

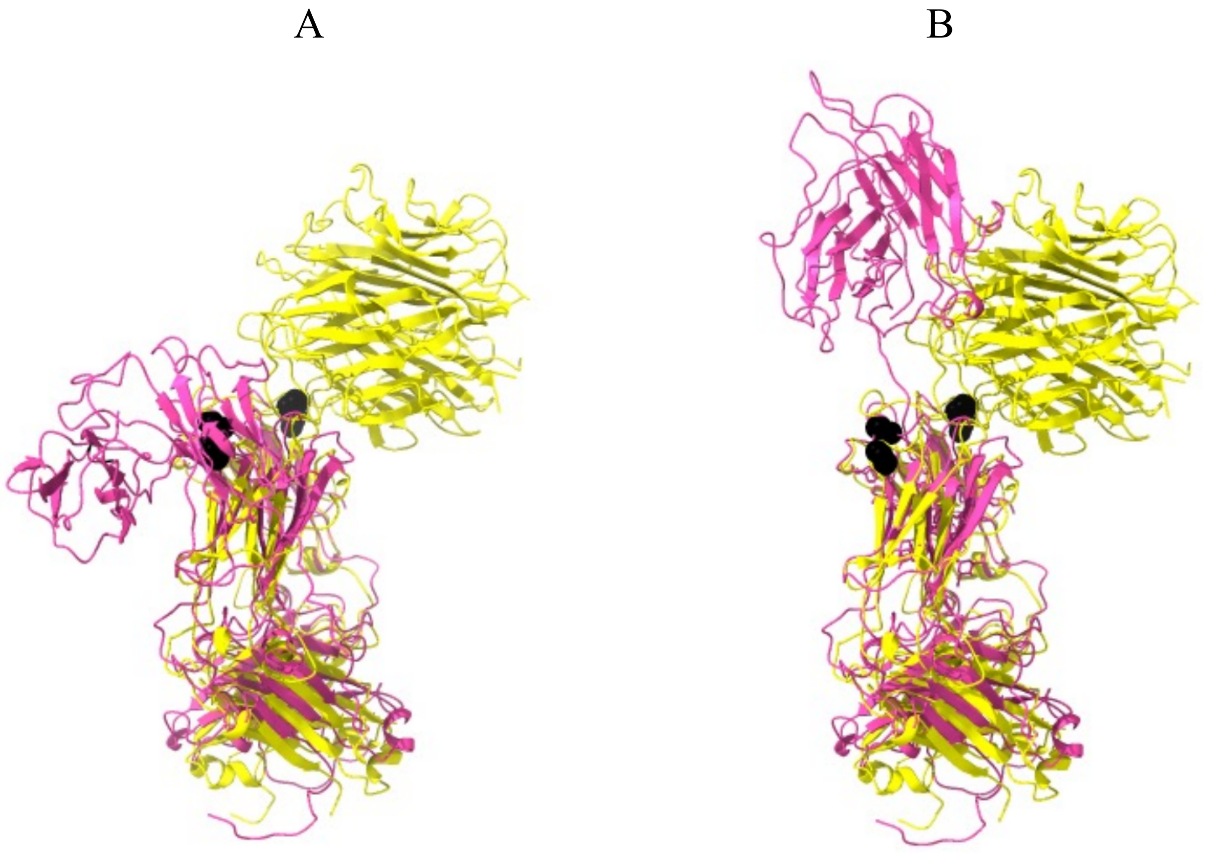

Supplementary Figure 3
Two of the IgM-Fc models (shown in pink), one partially bent (A) and one fully extended (B), chosen to fit the SAXS intensity plot for this protein, are each superposed on the structure of the complex (shown in yellow) of IgG-Fc bound to the "head" domain of C1q (PDB:6FCZ). The C1q head domain (yellow) is seen clearly at the top in panel A, while the IgG-Fc domains (Cγ2 and Cγ3, also in yellow) are superposed on the homologous IgM-Fc domains (Cμ3 and Cμ4, shown in pink). The Cμ2 domains of IgM-Fc (pink) can be seen bent to the left in panel A, and extended upwards in panel B. Both panels A and B are shown in the same orientation. In panel A, there is no clash between the Cμ2 domains (or any other part) of IgM-Fc (pink) and the head domain of C1q (yellow), and thus C1q is expected to bind to this bent conformation of IgM-Fc. Two key residues in the C1q binding site on IgM-Fc, Pro329 and Pro331 (refer text) are indicated as black spheres, two on each Cμ3 domain. In panel B, the Cμ2 domains of IgM-Fc (pink) in the fully extended conformation clash with the head domain of C1q (yellow), and thus C1q cannot bind to this conformation of IgM-Fc.

Supplementary Figure 4

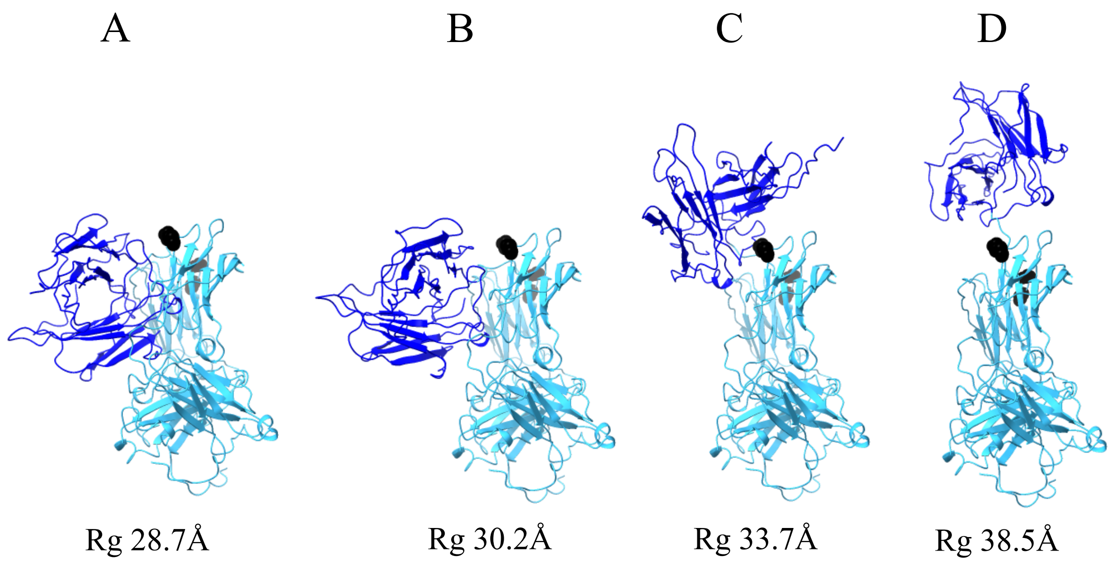

Supplementary Figure 4
The four models (A – D) selected to fit the SAXS intensity plot for IgY-Fc, shown in order of their increasing Rg values (28.7Å, 30.2Å, 33.7Å and 38.5Å), from acutely bent to fully extended. The Cυ2 domain pairs are coloured dark blue to clarify the bending. The fractional contribution of each conformation to the ensemble (respectively 6%, 25%, 34% and 35%), results in an average Rg value of 35.1Å, very close to the average Rg value of 35.6Å reported in Table 1. Two key proline residues in the C1q binding site of IgM (see Supplementary Figure 3 and refer text) are conserved in IgY, and are indicated here (black spheres on the Cυ3 domain at the front, grey spheres on the Cυ3 domain at the back). If C1q binding to IgY does occur, we expect the site to be accessible in conformations A and B, but not C and D.

References

1: Svergun D.I., Barberato C. and Koch M.H.J. (1995) CRYSOL - a Program to Evaluate X-ray Solution Scattering of Biological Macromolecules from Atomic Coordinates. J. Appl. Cryst. 28, 768-773. doi: 10.1107/S0021889895007047.
